# Prevalence of testicular disorders in dogs and cats in Fortaleza from 2020 to 2023

**DOI:** 10.1590/1984-3143-AR2024-0103

**Published:** 2026-03-20

**Authors:** Amanda Bricio Pereira de Andrade, Raul Kelvin Silveira Maia, Maria Eduarda do Carmo Moura, Herlon Victor Rodrigues Silva, Lúcia Daniel Machado da Silva

**Affiliations:** 1 Laboratório de Reprodução Animal, Faculdade de Veterinária, Universidade Estadual do Ceará – UECE, Fortaleza, CE, Brasil; 2 Universidade Federal do Ceará – UECE, Fortaleza, CE, Brasil

**Keywords:** epidemiology, reproduction, ultrasound, canine, feline

## Abstract

Considering the clinical importance and the frequent occurrence of several testicular pathologies, this study aimed to assess the prevalence of testicular disorders in dogs and cats and examine their association with species, breed, and age. In order to achieve this, ultrasound reports from the hospital’s system were utilized. The odds ratio was calculated for species and breed, while Pearson’s correlation test was used for age. A total of 1,282 dogs and 728 cats were examined. Of these, only the intact animals were considered susceptible to testicular conditions — 1,047 dogs (81.6%) and 218 cats (29.9%). Among dogs, 33.05% presented at least one testicular disorder, compared to 10.55% of cats. The most prevalent conditions were ectopia and degeneration, with neoplasia being frequently observed in canines. Other conditions identified included orchitis, fibrosis/microcalcification, cysts, hydrocele, monorchidism, torsion, and traumatic rupture. Canine species had a higher risk of testicular disorder and cryptorchidism. In dogs, age was strongly and positively related (p<0.001) to a higher prevalence of disorders. For general testicular disorders, poodle and pinscher breeds showed higher risks, while rottweilers had a lower risk compared to mixed-breed dogs. For ectopia, pinscher, shih-tzu, and poodle breeds had higher risks. Thus, it is evident that testicular , while less prevalent conditions deserve greater attention . In felines, the occurrence is less frequent and congenital conditions like ectopia and monorchidism are more common.

## Introduction

According to the Brazilian Institute of Geography and Statistics (IBGE), in 2019, approximately 46.1% of Brazilian households had at least one canine individual (IBGE, 2023a) and 19.3% had at least one feline (IBGE, 2023b). Subsequently, non-governmental research described a significant increase in these populations during the COVID-19 pandemic, especially felines. The state of Ceará, where this research was conducted, has the second highest percentage of households with at least one cat, as does its capital (IBGE, 2023b), and also has over 1 million households with at least one dog (IBGE, 2023a). Therefore, the social importance of these species is clear and yet there are no studies evaluating reproductive disorders in the population in the state.

Epidemiological studies are a fundamental starting point for assessing the importance and effectiveness of disease prevention actions in a population. The impact of the human population behavior should not be overlooked as part of the environmental factor directly linked to the history of diseases studied in veterinary epidemiology (Thrusfield, 2018). Additionally, the cultural impact of the COVID-19 pandemic on the human population and, consequently, on how they care for their pets should be considered. Thus, epidemiological results from different time periods and/or geographic areas may not be repeated in other locations at the present time. To date, no studies have assessed the prevalence of testicular disorders in companion animals in Ceará.

Ultrasonography is the preferred technique for characterizing testicular pathologies such as cryptorchidism and testicular neoplasms, and also aids in screening for other conditions related to enlargement of the scrotal area such as hydrocele, orchitis, epididymitis, and testicular torsion (Carvalho et al., 2014; Volta et al., 2014; Souza et al., 2017; Fulton, 2021), although it cannot distinguish malignant from benign lesions, this complementary exam is well-disseminated due to its speed, efficacy, safety, and low cost (Carvalho et al., 2014) and, considering its high acceptance by veterinarians and pet owners, it can be valued as a tool in the epidemiological study of a population.

The main testicular condition found in canine and feline species is cryptorchidism, a congenital defect in which the descent of one or both testicles fails to occur properly into the scrotum (Fulton, 2021). These ectopic testicles have a higher risk of developing other testicular pathologies such as neoplasia and torsion, affecting both canine (Khan et al., 2018) and feline (Little, 2011) species. Ultrasonography is a sensitive exam for locating ectopic testis, with up to 100% positive predictive value and 96.6% to 100% sensitivity based on their location (Felumlee et al., 2012). In dogs, testicular neoplasms are the second most frequent type of neoplasm, following only to skin neoplasms; however, the ultrasound exam is not the appropriate method for determining the degree of malignancy (Lawrence and Saba, 2012; Fulton, 2021).

Other common pathologies such as orchitis and testicular degeneration have been histologically described as common in both dogs and tomcats (Ortega‐Pacheco et al., 2006; Silva et al., 2022). Nevertheless, there are no studies evaluating the prevalence of this diagnostic suggestion through ultrasonographic examinations. In contrast, less common conditions such as testicular torsion and hydrocele are easily detected by ultrasonography (Fulton, 2021).

Considering the great importance and frequent involvement of testicles by a great variety of pathologies, the present study aimed to describe the prevalence of testicular disorders in dogs and cats attended at the Veterinary Hospital of the State University of Ceará between 2020 and 2023 and to verify the existence of a relationship between the development of these disorders and the factors: species, breed, and age.

## Methods

### Study design

Ultrasonographic reports were collected from exams performed at the Veterinary Hospital Professor Sylvio Barbosa Cardoso of State University of Ceará (UECE), located in Fortaleza, Brazil. This retrospective study covered the period from January 2020 to December 2023. Only reports referring to dogs and cats where the abdominal cavity and scrotal sac were evaluated were included. All patients had been previously examined by a veterinarian clinician at the mentioned hospital.

### Data collection

A database was created by adding the text described in each report into individual cells on Microsoft Excel Office 16. Data from each patient was tabulated using text scanning formulas for the respective columns: ID number, species, breed, sex, age, reproductive status and testicular evaluation.

### Ultrasound interpretation

Ultrasonographic description of the collected reports followed a specific textual model to ensure standardized evaluation of the necessary criteria. When referring to each testicle, a standard textual model was used to describe normal findings : “in usual topography, with regular contours, standard echogenicity and homogeneous echotexture. Preservation of the hyperechoic mediastinal line. Measured 1.5 cm in width and 1.2 cm in height. Standard sonographic appearance.” Whenever any of the structures were not visible, whether the patient was neutered or not, the textual model was completely replaced with the expression: “not visible” or “not visible(s).”

In absence of normality and non-visibility descriptions, the text was fully evaluated, and the clinical suspicion equivalent to the ultrasonographic description was defined. In cases where more than one condition occurred simultaneously, all of these were described and the patient was included for prevalence determination of all present reproductive conditions.

Systematic assessment was performed to classify the testicles as: not visible, visible with normality, and visible with testicular disorder defined by a trained operator. When an abnormality was presented in one or both testicles, the patient was classified as with testicular disorder and included for prevalence determination of the respective reproductive conditions.

### Statistical analysis

The evaluated risk factors to the development of overall testicular conditions were: species, breed and age at diagnosis. Species and breed were also evaluated as risk factors for cryptorchidism. In order to avoid significant statistical biases when studying breed as a risk factor, only breeds with at least 15 patients were selected.

When assessing the prevalence of testicular conditions in each species, it is important to reiterate that the study of real prevalence should consider only the population susceptible to the development of the condition in question. Thus, the total male population of the species was considered for the calculation of overall prevalence, and the population of intact males of the species was considered to calculate the corrected prevalence and to assess risk factors for testicular conditions. This data was expressed in percentage and its confidence interval data was calculated for a 95% confidence level.

Odds ratio with 95% confidence intervals were used to evaluate species and breed as risk factors for the development of overall testicular conditions and for cryptorchidism. The reference comparative group was feline (for species) and mixed-breed (for breed) patients. For age at diagnostic, Pearson’s correlation test was used to determine the coefficient of determination between patient’s age and prevalence of non-congenital testicular conditions, with p-values <0.05 being considered statistically significant. Data curation and formal statistical analyses were made in Power BI Desktop and Jamovi 2.3.28 programs.

### Ethical aspects

This study adhered to the ethical authorizations required for conducting scientific research involving animals with the approved of Ethics Committee for Animal Experimentation of UECE under number 05167487/2023. Used data were collected at the Veterinary Hospital Professor Sylvio Barbosa Cardoso of UECE, in Fortaleza, Brazil from January 2020 to December 2023.

## Results

A total of 2,010 males were examined with ultrasonography from January 2020 to December 2023, of which 1,282 were dogs and 728 were cats. Regarding reproductive status, only intact patients are considered susceptible to testicular conditions: 1,047 dogs (81.6% of total dogs) and 218 tomcats (29.9% of total cats). Therefore, 235 neutered dogs (18.33%) and 510 neutered cats (70.05%) were excluded from the study.

The total percentage of patients with at least one suspicion of testicular disorder was 32.92% of susceptive dogs and 10.55% of susceptive cats, as demonstrated by the corrected prevalence in Tables 1 and 2, respectively. All testicular disorder suspicions, except for monorchidism and traumatic parenchymal rupture were more prevalent in dogs compared to tomcats. Conditions found exclusively in dogs in this study were: testicular mass, cysts in the parenchyma, hydrocele, and testicular torsion.

**Table 1:** Number of dogs with each testicular disorder indicated by B-mode ultrasonographic examination, along with the respective overall and adjusted prevalence and the confidence interval of the adjusted prevalence for the population treated at HVSCB from 2020 to 2023.

**Testicular acquired affections**	**Affected dogs**	**Overall prevalence**	Corrected prevalence**	**IC** ^*^ **- 5%**	IC* - 95%
Testicular degeneration	101	7.88%	9.65%	7.86%	11.43%
Testicular mass	80	6.24%	7.64%	6.03%	9.25%
Orchitis	73	5.69%	6.97%	5.43%	8.51%
Mineralization/Fibrosis spots	34	2.65%	3.25%	2.17%	4.32%
Cyst	16	1.25%	1.53%	0.79%	2.27%
Hydrocele	12	0.94%	1.15%	0.50%	1.79%
Testicular torsion	3	0.23%	0.29%	0***	0.61%
Traumatic parenchymal rupture	2	0.16%	0.19%	0^***^	0.46%
**Testicular congenital affections**	**Affected dogs**	**Overall prevalence**	**Corrected prevalence** ^**^	IC* - 5%	IC* - 95%
Cryptorchidism	152	11.86%	14.52%	12.38%	16.65%
Monorchidism	4	0.31%	0.38%	0.01%	0.76%
**Total affected patients**	**346**	**26.99%**	**33.05%**	**30.20%**	**35.90%**

* IC: confidence interval; **Based on intact males; ***Negative values.

**Table 2:** Number of cats with each testicular disorder indicated by B-mode ultrasonographic examination, along with the respective overall and adjusted prevalence and the confidence interval of the adjusted prevalence for the population treated at HVSCB from 2020 to 2023.

**Testicular congenital affection**	**Affected cats**	**Overall prevalence**	**Corrected prevalence** ^**^	**IC** ^*^ **- 5%**	IC* - 95%
Cryptorchidism	12	1.65%	5.50%	2.48%	8.53%
Monorchidism	5	0.69%	2.29%	0.31%	4.28%
**Testicular acquired affection**	**Affected cats**	**Overall prevalence**	Corrected prevalence**	IC* - 5%	IC* - 95%
Testicular degeneration	6	0.82%	2.75%	0.58%	4.92%
Orchitis	4	0.55%	1.83%	0.05%	3.62%
Mineralization/Fibrosis spots	1	0.14%	0.46%	0***	1.36%
Traumatic parenchymal rupture	1	0.14%	0.46%	0^***^	1.36%
**Total affected patients**	**23**	**3.16%**	**10.55%**	**6.47%**	**14.63%**

Feline population had high neutering rates (70.05%), limiting direct comparisons. *IC: confidence interval; **Based on intact males; ***Negative values.

Patients with suspected monorchidism had their clinical history carefully evaluated to exclude those whose second testicle had been previously removed, resulting in a more accurate prevalence. Thus, two patients (one dog and one tomcat) were found to have exclusively removed one normal testicle and retaining an ectopic testicle, and one dog had exclusively removed an ectopic testicle while retaining the normal testicle.

Degeneration, hydrocele e parenchyma cysts were assessed for the presence of other testicular disorders. Then, 64.4% (65/101) of cases of testicular degeneration were associated with suspicions of orchitis, ectopia, or testicular masses of the contralateral testicle. Also, out of the twelve cases of hydrocele in this study, four (33.3%) were associated with the presence of testicular mass, two (16.7%) were associated with testicular torsion, and six (50.0%) did not show any other associated ultrasound abnormalities. Regarding parenchyma cysts, four of the seventeen dogs (23.5%) had associated ultrasound-detected testicular mass and, additionally, the average age of diagnosed animals was 10 years.

## Species

The canine species had higher odds of developing testicular conditions (OR = 4.18, 95% CI: 2.67–6.57) and cryptorchidism (OR = 2.92, 95% CI: 1.59–5.35) compared to felines

## Age

**Figure 1:**
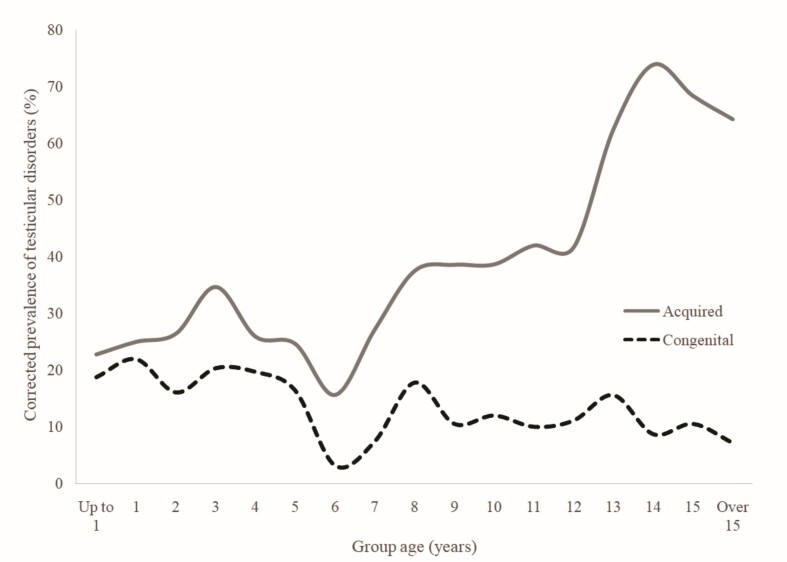
Corrected prevalence of congenital and acquired testicular disorders by group age for canine population treated at HVSCB from 2020 to 2023.

At the age of diagnostic, 64.6% (103/164) of patients with ectopic testicle were over 2 years old, and 26.2% (43/164) were over 7 years old. To reduce bias related this condition`s congenital nature, the study was conducted only on acquired testicular conditions in the population not affected by ectopic testicles or monorchidism, as described in Table 3 and Figure 1.

**Table 3:** Number and corrected prevalence of dogs affected congenital and acquired testicular disorders for the population treated at HVSCB from 2020 to 2023.

**Age group**	**Number of intact dogs**	**Dogs with congenital testicular disorders**	**Dogs with acquired testicular disorders**
Up to 11 months	101	19 (18.81%)	23 (22.77%)
1 year	100	22 (22%)	25 (25%)
2 years	87	14 (16.09%)	23 (26.43%)
3 years	98	20 (20.4%)	34 (34.69%)
4 years	81	16 (19.75%)	21 (25.92%)
5 years	73	12 (16.43%)	18 (24.65%)
6 years	64	2 (3.12%)	10 (15.62%)
7 years	81	6 (7.4%)	22 (27.16%)
8 years	56	10 (17.85%)	21 (37.5%)
9 years	57	6 (10.52%)	22 (38.59%)
10 years	75	9 (12%)	29 (38.66%)
11 years	50	5 (10%)	21 (42%)
12 years	36	4 (11.11%)	15 (41.66%)
13 years	32	5 (15.62%)	20 (62.5%)
14 years	23	2 (8.69%)	17 (73.91%)
15 years	19	2 (10.52%)	13 (68.42%)
Over 15 years	14	1 (7.14%)	9 (64.28%)
**Total**	**1047**	**155 (14.8%)**	**194 (21.68%)**

**Figure 2:**
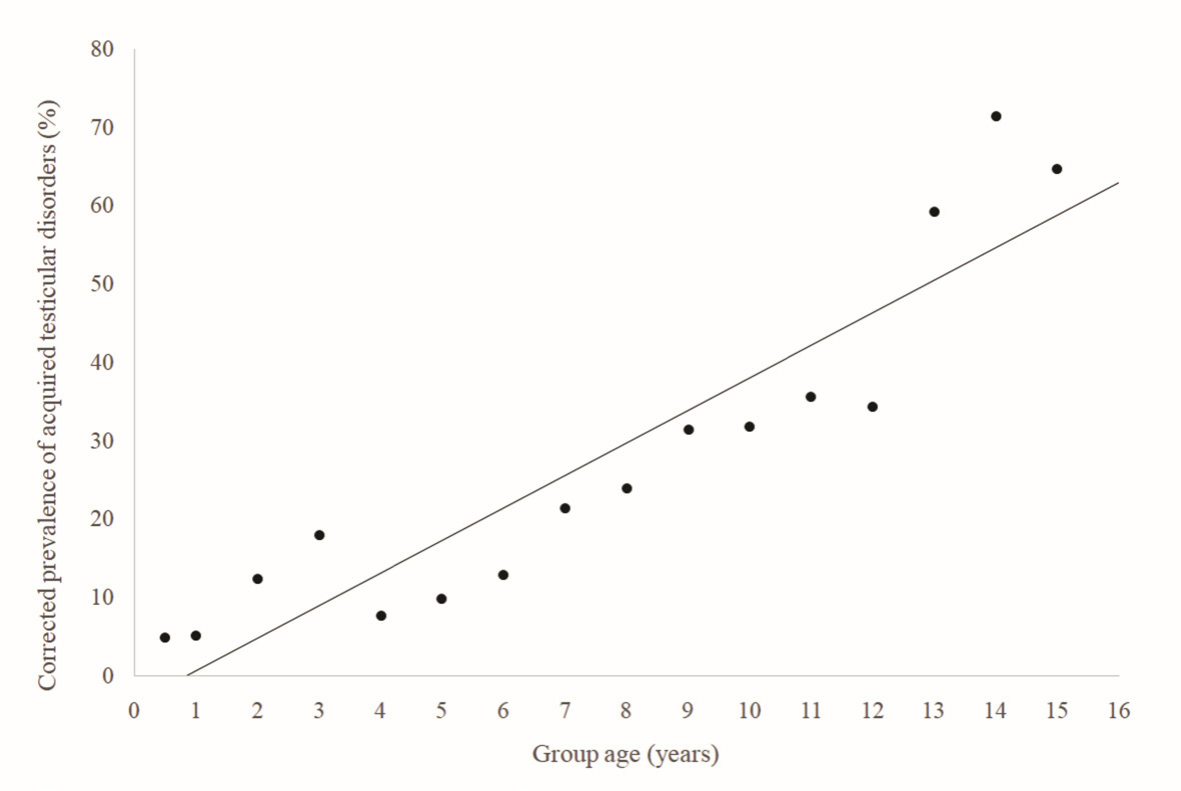
Relationship between the prevalence of acquired testicular conditions and age (in years) with the corresponding trend line in unneutered dogs without congenital disorders treated at HVSCB from 2020 to 2023.

Using Pearson’s correlation test for the frequency of testicular acquired conditions in the canine species, an R of 0.929 (very strong correlation) was obtained with p0.001 (highly significant), under normally distributed data. The corrected prevalence of these conditions reached above 20% from age 7, above 35% from age 10 and above 60% from 13 years old, as shown in Figure 2. When evaluating all patients aged 7 years and older as a group, 42.66% of the patients were affected by some testicular disorder.

When assessing the relationship between age and testicular conditions in felines, the population did not have a sufficient distribution of acquired conditions to evaluate age as a risk fator, as shown in Table 4.

**Table 4:** Number and corrected prevalence of tomcats affected congenital and acquired testicular disorders for the population treated at HVSCB from 2020 to 2023.

**Age group**	**Number of tomcats**	**Cats with congenital testicular disorders**	**Cats with acquired testicular disorders**
Up to 11 months	45	2 (4.44%)	1 (2.22%)
1 year	44	4 (9.09%)	0 (0%)
2 years	35	4 (11.42%)	0 (0%)
3 years	22	3 (13.63%)	0 (0%)
4 years	20	1 (5%)	1 (5%)
5 years	8	0 (0%)	1 (12.5%)
6 years	9	2 (22.22%)	1 (11.11%)
7 years	6	0 (0%)	0 (0%)
8 years	9	0 (0%)	2 (22.22%)
9 years	4	0 (0%)	0 (0%)
Over 9 years	16	1 (6.25%)	0 (0%)
**Total**	**218**	**17 (7.79%)**	**6 (2.75%)**

## Breed

**Figure 3:**
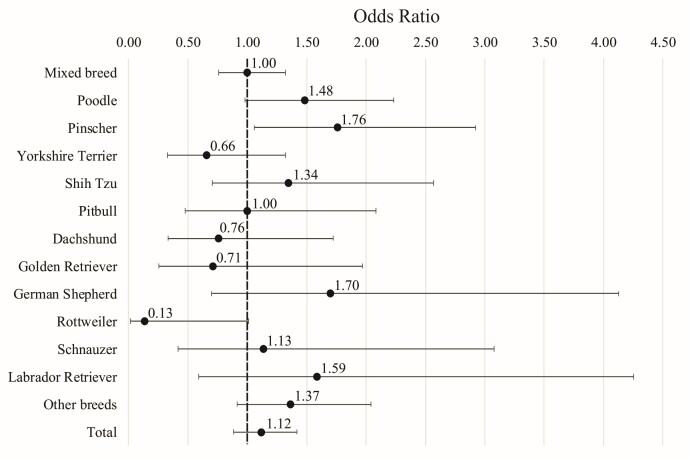
Odds ratio and confidence interval for testicular conditions in canine breeds treated at HVSCB from 2020 to 2023.

The odds ratio for testicular conditions by canine breed is described in Figure 3. Pinscher breed had 1.6 higher risks for developing testicular conditions compared to mixed-breed dogs, respectively. When considering the confidence interval of 90%, rottweiler individuals presented 0.3 lower risk and Poodle presented 1.5 higher risk when compared to mixed-breeds.

For feline population, there were not enough individuals of each breed to perform the evaluation. The distribution was 209 (96.5%) mixed-breed individuals, 5 (2.75%) Siamese, and 4 (2.29%) Persian.

**Figure 4:**
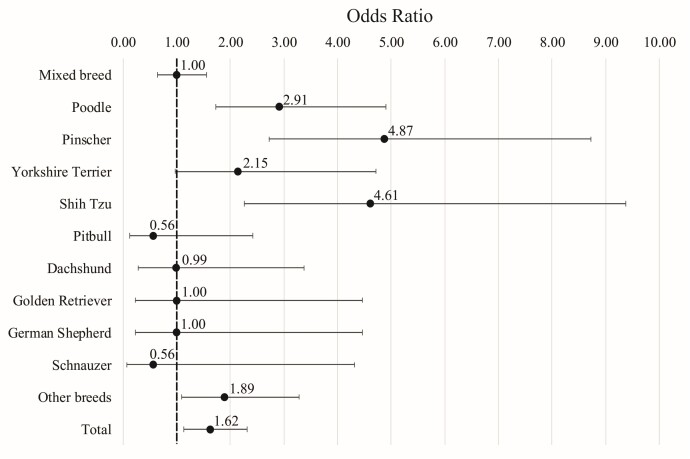
Odds ratio and confidence interval for cryptorchidism in canine breeds treated at HVSCB from 2020 to 2023.

Among testicular conditions, ectopia was the most prevalent. Canine breeds with more than 15 intact patients in the study, and which had a higher frequency of cryptorchidism, were: Pinscher (33.8%), Shih-Tzu (32.6%), and Poodle (23.2%). Patients of these three breeds had higher risks of ectopic testicle compared to mixed-breed dogs, as demonstrate in Figure 4. Additionally, when considering all pure breed patients versus mixed-breed patients, there was a 1.61 higher risk of testicular disorders in purebred individuals.

## Testicular location

Regarding the location of ectopic testicles in the studied patients, a higher frequency of unilateral right involvement and inguinal location was found in dogs, as shown in Tables 5 and 6. In felines, the low frequency of this affection did not allow for comparative evaluations.

**Table 5:** Number and frequency of canine and feline patients with ectopic testicle according to the affected side in the population treated at HVSCB from 2020 to 2023.

**Ectopic testicle**	**Dogs**	**Cats**
Unilateral Left	37 (24.34%)	4 (33.33%)
Unilateral Right	75 (49.34%)	4 (33.33%)
Unidentified	8 (5.26%)	3 (25%)
Bilateral	32 (21.05%)	1 (8.33%)
**Total**	**152 (100%)**	**12 (100%)**

**Table 6:** Number and frequency of canine and feline patients with ectopic testicle according to testicle topography in the population treated at HVSCB from 2020 to 2023.

**Ectopic location**	**Dogs**	**Cats**
**Inguinal**	**86 (56.57%)**	**5 (41.66%)**
Left	16 (18.6%)	1 (8.33%)
Right	51 (59.3%)	1 (8.33%)
Bilateral	17 (19.76%)	1 (8.33%)
Unidentified	2 (2.32%)	2 (16.66%)
**Intra-abdominal**	**34 (22.36%)**	**2 (16.66%)**
Left	10 (6.57%)	1 (8.33%)
Right	15 (9.86%)	1 (8.33%)
Bilateral	6 (3.94%)	0 (0%)
Unidentified	3 (1.97%)	0 (0%)
**Subcutaneous**	**25 (16.44%)**	**5 (41.66%)**
Left	11 (7.23%)	2 (16.66%)
Right	9 (5.92%)	2 (16.66%)
Bilateral	5 (3.28%)	0 (0%)
Unidentified	0 (0%)	1 (8.33%)
**Mixed**	**7 (4.6%)**	**0 (0%)**
Inguinal and intra-abdominal	2 (1.31%)	0 (0%)
Inguinal and subcutaneous	1 (0.65%)	0 (0%)
Subcutaneous and intra-abdominal	1 (0.65%)	0 (0%)
Unidentified	3 (1.97%)	0 (0%)
**Total**	**152 (100%)**	**12 (100%)**

## Discussion

Studies describing the frequency of testicular conditions, except for cryptorchidism in dogs and cats and neoplasms in dogs, are scarce. These studies are limited to epidemiological data with small sample sizes or were published over two decades ago. Less common conditions have received little to no prevalence assessment in the literature. Thus, this work intends to discuss not only the most frequent conditions but also explore studies reporting other conditions and provide new epidemiological data for both species. In this context, the substantial sample size of the present study strengthens the robustness of the analysis and contributes to advancing current knowledge in the field.

Regarding the population in question, the feline species had a much higher frequency of patients who had been previously neutered (70.1%), which may be related to a greater tendency of feline owners to perform orchiectomy compared to dog owners (18.4%). Thus, the corrected prevalence of testicular conditions in this species has become more discrepant from the general prevalence compared to the canine species.

Moreover, the feline species showed a significantly lower risk of testicular conditions and specifically of cryptorchidism. Although literature describes that most lesions of the male reproductive tract in dogs and cats are reported in dogs (Foster, 2012), no previous studies have directly compared the risk between these two companion species within the same geographic population.

When analyzing the three most prevalent testicular conditions in this study, it is important to consider the polygenic factors intrinsically associated with cryptorchidism and neoplasms, as well as the heightened predisposition for degenerative changes and tumour development in ectopic testes (Little, 2011; Carvalho et al., 2014). Additionally, canine species show a higher overall predisposition to neoplasia compared to felines (Gupta et al., 2024). A possible explanation for the lower incidence of testicular disorders in felines may be the reduced presence—or lower expression—of genes related to the main testicular pathologies in cats.

Recent epidemiological studies describing all testicular conditions observed in a sample of a population are scarce. In dogs, the most recent study found by the authors was by Ortega‐Pacheco et al. (2006), which described a frequency of testicular conditions affecting 42.5% of the 318 unneutered dogs studied, higher than that found in the present study. In tomcats, the only study found was by Silva et al. (2022), which described histopathological involvement in 80% of testicles, but with a sample size of only 25 individuals.

Ectopic testis, or cryptorchidism, was the most prevalent condition in both species of this study, which is an expected result considering it as the most common congenital condition in these species (Little, 2011; Carvalho et al., 2014). According to Felumlee et al. (2012), ultrasonographic examination is the routine tool for diagnosing ectopic testis, with specificity reaching 100% under adequate conditions and sensitivity ranging from 96.6% to 100% depending on the testis location. Older studies, such as Wolverson et al. (1983), found lower sensitivity; however, it should be noted that these data are outdated due to the significant and constant technological advancements in ultrasonographic equipment.

Most studies describing the frequency of this condition were published over 30 years ago. After this period, four authors reported the percentage of cryptorchid dogs, ranging from 2.1% to 6.8% (Yates et al., 2003; Ortega‐Pacheco et al., 2006; Gubbels et al., 2009; Cho et al., 2025). Yates et al. (2003) and Cho et al. (2025) analysed populations of 3,518 and 5,476 dogs undergoing elective orchiectomy, respectively, whereas Gubbels et al. (2009) evaluated data from over 10,000 litters across 12 breeds.

In tomcats, prevalences ranging from 1.12% to 1.3% were reported in populations of 3,806 (Yates et al., 2003) and 11,559 (Cho et al., 2025) cats underwent elective orchiectomy and by Wallace and Levy (2006) when including total male and female populations in the calculation, underestimating the prevalence of this condition by considering individuals not susceptible to testicular conditions.

In both species, higher frequencies of cryptorchidism were observed in the present study, raising the question of whether the population in question has a higher frequency of genes linked to the occurrence of this condition compared to other previously studied populations.

Testicular masses were observed only in dogs in this study. This disorder could not be diagnosed with specificity when identified by ultrasonographic examination. The most frequent possibility in dogs, particularly in older ages, is that it is related to a neoplastic process (Carvalho et al., 2014; Volta et al., 2014). Besides neoplastic processes, differential diagnoses for images compatible with testicular mass include: hematomas, abscesses, and areas of necrosis or regenerative lesions, all previously described as rare in dogs (Johnston et al., 1991; Pugh and Konde, 1991).

The canine species has a higher frequency of testicular masses compared to other domestic species and humans (Baba and Câtoi, 2007; North and Banks, 2009). In humans, testicular cancer is the most common neoplasm among men aged 15–40 years but remains rare, representing about 1% of adult neoplasms (Giona, 2022). In bulls, such cases are also uncommon and mostly reported as isolated occurrences (Butkiewicz et al., 2025). In unneutered dogs, testicles are the second most affected organ by neoplasms, affecting between 6% and 12% of the population, only behind skin neoplasms (Lawrence and Saba, 2012). On the other hand, testicular neoplasms are rare in tomcats. Previously, Silva et al. (2022) evaluated histopathological changes in 25 feline testicles, and Cotchin (1957) evaluated 464 feline neoplasms, neither finding any neoplastic cases. Thus, the results of this study for both species align with expectations.

In this study, the presence of ultrasonographic signs linked to suspected testicular degeneration was the second most frequent condition in both species. Ultrasonographic examination is not capable of specifying the microscopic processes defined as degeneration; however, this condition is one of the most common causes of low fertility in males of domestic species, having multiple etiological factors and commonly associated with senility, orchitis, cryptorchidism, and contralateral testicular neoplasia (Foster, 2012; Matoon et al., 2014; Volta et al., 2014). In this study, 64.4% of the cases of degeneration were associated with suspected orchitis, ectopia, or neoplasia of the contralateral testis.

In dogs, Ortega‐Pacheco et al. (2006) found a prevalence of 15.1% of testicular degeneration, a higher frequency compared to the study in question. However, the low sensitivity of ultrasonographic examination, especially in the early stages of the condition, should be considered, potentially underdiagnosing the actual prevalence of degenerative processes detectable by microscopy.

In cats, Silva et al. (2022) evaluated histopathological changes in 50 testicles, finding 14% with degeneration of moderate to severe intensity, meaning they had macroscopic detectable signs. Although the referred study lacks epidemiological methodology, it demonstrates that it is common for the feline testis to be affected by degenerative microscopic processes, consistent with the findings of this study.

In canines, there was also a high prevalence of characteristics related to testicular inflammation (6.97%), a rare condition in tomcats (0.55%). It has been previously described that orchitis is more common in dogs compared to tomcats (Domingos and Salomão, 2011); however, no studies were found defining the frequency of this condition in populations of these species.

This study provides novel epidemiological data on less frequently reported testicular alterations in both dogs and cats, highlighting its clinical relevance in the differential diagnosis of testicular abnormalities in these species. Lower prevalences in both species were found for the following changes: presence of hyperechoic points, traumatic parenchymal rupture, and monorchidia. Notably, all cases of testicular parenchymal rupture had this finding correlated with a history of recent trauma. occurrence in both dogs and cats.

The ultrasonographic presence of hyperechoic points in parenchymal organs is commonly related to areas of fibrosis or mineralization. Testicular parenchymal fibrosis is a process directly linked to aging in dogs (Tesi et al., 2020), and mineralization spots may be related to micronodular neoplasms or dystrophic mineralizations such as endocrine or uremic (Cardoso et al., 2019). In tomcats, interstitial fibrosis has been described in 30% of testicles evaluated by histopathology, with 91.7% of the cases associated with testicular degeneration. This same study described one testicle (2%) affected by mineralization (Silva et al., 2022). Due to the essentially microscopic nature of fibrosis, it is expected that this lesion would be less detectable by ultrasonography. Therefore, to obtain a true distinct prevalence of these conditions, the authors suggest specific histopathological evaluations with an epidemiological focus.

Monorchidia is a rare congenital condition in both dogs and tomcats, with higher prevalence in pure breed patients (de la Puerta and Baines, 2012). In dogs, reported cases are rare, and there is no estimated prevalence in the literature even for pure breed patients, with the result of this study being a pioneer in describing of the frequency of this condition in dogs. In felines, the prevalence of this condition has been previously described as 0.1% (Millis et al., 1992). The result of this study may be overestimated, considering the high rate of neutered cats in this population and the constant indication for ultrasound examination by the veterinarian clinician to locate ectopic testicles prior to orchiectomy because of its high sensibility (Felumlee et al., 2012).

Other low-prevalence conditions found exclusively in dogs were testicular torsion, cyst, and hydrocele. To the authors' knowledge, this is the first study describing the prevalence of these conditions in dogs.

Testicular torsion, although no previous prevalence has been described, is not uncommon in dogs, with several reports cited by Raisi and Davoodi (2022). In tomcats, only one report was found (Giuliano, 2013), making it an especially rare condition in the species. Ultrasonographic examination can provide sufficient evidence of this condition, regardless of the testicular location (Raisi and Davoodi, 2022).

In the current study, hydrocele cases associated with neoplasia (Pugh and Konde, 1991) or vascular conditions (Draheim et al., 2022) in dogs have been described. Regarding the presence of cysts in the testicular parenchyma, reports not associated with neoplasia are especially rare.

Regarding age as a risk factor for the conditions found, considering the congenital nature of ectopic testis and the age distribution of this condition found in the study, a significant bias is expected in the assessment of age as a factor. This is because many cryptorchid patients are diagnosed at older ages, or when diagnosed young, the owner may choose not to perform orchiectomy. Furthermore, ectopic testis is a risk factor already defined in the literature for the development of other testicular pathologies (Little, 2011; Matoon et al., 2014), especially in the canine species (Yates et al., 2003; Khan et al., 2018). Thus, the study excluded patients with congenital conditions (cryptorchidism and monorchidia) from this evaluation to avoid skewing the data and affecting the results related to the age factor.

The positive correlation between age and the prevalence of testicular conditions found in this study is consistent with the expectations of the literature. In addition to the increased risk of developing testicular masses, other testicular conditions have been reported as more common in older canines. Ortega‐Pacheco et al. (2006) described higher prevalence of testicular degeneration in dogs over six years old. Testicular torsion also becomes more common in older patients, particularly between eight and ten years of age (Raisi and Davoodi, 2022). In this study, testicular involvement starting from seven years of age was very frequent, affecting more than 20% of the population in the respective age groups and reaching over 60% in patients aged 13 years and older. Thus, it should be highlighted that not only elderly dogs but also middle-aged adults are commonly affected by testicular conditions.

Regarding breed predisposition, three breeds showed a significantly higher risk for cryptorchidism (poodle, pinscher, shih-tzu) and one for general testicular conditions (pinscher) compared to mixed-breed patients. Various poodle breeds (miniature, toy, standard) have been described as having a higher predisposition to both testicular testicular masses and cryptorchidism. For the pinscher, no other studies confirming these breed predispositions were found (Felumlee et al., 2012; Gough et al., 2018). Regarding the shih-tzu, previous studies also described a higher predisposition to cryptorchidism (Yates et al., 2003).

When evaluating pure breed patients as a single group, a higher predisposition to cryptorchidism was observed. Hayes et al. (1985) previously described a higher predisposition to cryptorchidism in smaller breeds, which accounted for 67.12% (392/584) of the pure breed patients in the studied population. Considering that 40.82% (238 of 583) of the male pure breed patients in the studied population were part of the three breeds that showed predisposition, this result is consistent with previous data reported in the literature.

These results highlight the importance of targeted preventive and diagnostic strategies for at-risk breeds such as poodles, pinschers, and shih-tzus. Early screening protocols—such as thorough testicular palpation during pediatric veterinary visits and the use of ultrasound in ambiguous cases—may aid in the early detection of cryptorchidism or other testicular anomalies. Moreover, these results should be used to raise awareness among breeders and veterinarians, thereby contributing to more timely surgical castrations and reducing the risk of degenerative changes and neoplasms, especially in older, intact males. Finally, when evaluating the distribution of ectopic testicles found in this study, a greater tendency for inguinal location and right unilateral involvement has been previously described in dogs (Yates et al., 2003; Tannouz et al., 2019). In tomcats, the inguinal location and unilateral involvement have also been shown to be more frequent; however, both testes seem to be equally affected (Yates et al., 2003; Little, 2011), similar to the results of this study. Therefore, it must be reiterated that, as with non-reproductive pathologies, the feline species cannot be essentially equated with the canine when discussing testicular disorders.

Thus, the presence of ectopia and degeneration were the most common disorders for both species, with much higher prevalence in dogs when compared to tomcats. Presence of testicular masses in dogs and monorchidism in tomcats were also common. Other clinically significant conditions should not be overlooked, with this study being the pioneer in describing the epidemiological prevalence of orchitis and testicular parenchymal rupture by trauma in both species and parenchymal cysts, hydrocele and monorchidia in dogs. Among the predisposing factors, adult dogs from seven years old and poodle, pinscher and shih-tzu breeds show a higher predisposition to testicular conditions. Cryptorchidism is more frequently observed in the inguinal region for both species and affecting the right testicle in canine species.

## Conclusion

Testicular conditions are highly prevalent in canine species and are commonly diagnosed through ultrasound examination. In cats, testicular involvement is less common compared to dogs. In both species, ectopia and degeneration are the most prevalent testicular condition. Adult dogs from seven years old and of poodle, pinscher and shih-tzu breeds show a higher predisposition to testicular affections. Thus, routine testicular ultrasound screening is recommended for intact dogs over 7 years of age.
